# Clinical trial on the pharmacokinetics, pharmacodynamics and safety of tolvaptan in healthy Chinese males: an open-label, single and multiple dosage, parallel group study

**DOI:** 10.3389/fphar.2025.1713702

**Published:** 2025-11-21

**Authors:** Hongzhong Liu, Tao Liu, Ming Liu, Xin Zheng, Wen Zhong, Qian Zhao, Ji Jiang, Pei Hu, Yishi Li

**Affiliations:** 1 Clinical Pharmacology Research Centre, Peking Union Medical College Hospital, Chinese Academy of Medical Sciences & Peking Union Medical College; Beijing Key Laboratory of Clinical PK & PD Investigation for Innovative Drugs, Beijing, China; 2 Key Laboratory of Clinical Trial Research in Cardiovascular Drugs, Ministry of Health, Cardiovascular Institute and Fuwai Heart Hospital, Chinese Academy of Medical Sciences (CAMS), Beijing, China

**Keywords:** tolvaptan, pharmacokinetics, pharmacodynamics, safety, urine volume

## Abstract

**Background:**

To determine the pharmacokinetics (PK), pharmacodynamics (PD) and safety of tolvaptan in healthy Chinese males.

**Methods:**

Three separate clinical trials were carried out on healthy Chinese males aged 18–45 years. Fifty received a single dose of tolvaptan of 7.5, 15, 30, 60 and 120 mg. In addition, 36 received multiple doses of 7.5, 30 and 60 mg once a day for 7 days. The primary outcomes measured were the PK parameters of tolvaptan and its two metabolites (DM-4103, DM-4107). Secondary endpoints included serum electrolytes, urine volume and water intake, which were monitored as pharmacological indicators. The safety profile was also evaluated in detail.

**Results:**

After the administration of a single dose of tolvaptan, dose proportionality was observed for the area under the concentration-time curve (AUC) from 7.5 mg to 120 mg, but not for the maximum plasma concentration (C_max_). The mean (SD) values of C_max_ from 7.5 mg to 120 mg were 69.8 (24.0), 102.0 (17.4), 245.5 (82.9), 323.9 (141.4), and 587.1 (364.0) ng/mL, respectively. Following multiple dose administration of 7.5, 30 and 60 mg tolvaptan once a day for 7 days, dose proportionality for the AUC was observed. The steady-state concentration was reached within 7 days of administration once a day. The accumulation ratios of AUCs were 1.2, 1.2 and 1.2 for the 7.5, 30 and 60 mg doses, respectively. Within the dosage range 7.5–120 mg, urine volume increased with dose after both single and multiple oral administrations of tolvaptan. No clinically significant changes in serum electrolytes (K^+^, Na^+^, Cl^−^, Mg^2+^) were detected following oral administration of 7.5 mg tolvaptan. The most common adverse events after single and multiple doses of tolvaptan were thirst, dry mouth and pharyngeal discomfort, which were known and predictable effects of tolvaptan.

**Conclusion:**

Tolvaptan demonstrated good tolerability and efficacy after single doses up to 120 mg and multiple doses up to 60 mg per day for 7 days. Dose proportionality was observed for AUC from 7.5 mg to 120 mg, but not for C_max_. Similar PK profiles were observed between single and multiple doses with slight accumulation.

**Clinical Trial Registration:**

https://clinicaltrials.gov/, identifier NCT07166796, NCT07166783 and NCT07166887.

## 1 Introduction

The United States Food and Drug Administration have approved the use of tolvaptan to treat hypervolemic and euvolemic hyponatremia, including in patients with congestive heart failure (CHF) and inappropriate antidiuretic hormone syndrome (Zmily et al., 2011). The initial dose is 15 mg once a day, and can be increased to a maximum of 60 mg once a day to increase serum Na^+^ to appropriate concentrations (Yi et al., 2012). (Tzoulis et al., 2023). These actions form the basis of its effects in treating patients with edema and/or hyponatremia.

The pharmacokinetic (PK) and pharmacodynamic (PD) profiles of tolvaptan have been extensively studied in hyponatremic patients under different pathogenic conditions. Following approval, two randomized, double-blind, placebo-controlled, ascending single-dose studies in predominantly Caucasians revealed a dose-dependent increase in cumulative urine output at 72 h after doses ranging from 60 mg to 480 mg. Despite similar cumulative urine output and aquaretic effects at all doses within the first 12 h after dosing, dose-limiting toxicity did not occur (Gheorghiade et al., 2003; Schrier et al., 2006). In addition, a study that evaluated doses <60 mg in 42 subjects showed that the mean half-life (t_1/2_) of tolvaptan increased from 3.3 h (15 mg) to 11.4 h (120 mg). Peak concentrations occurred within 2–3 h, with complete clearance within 24 h. Multiple dosing did not result in drug accumulation or differences in urinary excretion (Kim et al., 2011).

In healthy adults, after single oral doses of tolvaptan (15–480 mg), the terminal phase elimination half-life (t_1/2, z_) increased with higher doses, from approximately 3 h for a dose of 15 mg to 12 h for 120–480 mg doses (Shoaf et al., 2012b). Tolvaptan is mainly absorbed from the upper gastrointestinal tract at low doses, with elimination processes dominating the terminal concentration curve. At higher doses, absorption continues throughout the gastrointestinal tract, which affects the terminal phase rate of decline. The maximum plasma concentration (C_max_) exhibited a less than dose-proportional increase from 30 mg to 240 mg, plateauing at 240–480 mg (Shoaf et al., 2007). Despite variations in absorption, the area under the concentration-time curve (AUC) increased proportionally with the dose, and the apparent clearance (CL/F) remained constant for single doses between 30 mg and 480 mg (Kim et al., 2011). It has been shown that tolvaptan did not accumulate when administered once a day and that its PK properties were not influenced by age, sex or race (Shoaf et al., 2017).

In the landmark SALT clinical trials, which investigated tolvaptan for the treatment of hyponatremia in patients with chronic heart failure, cirrhosis or the syndrome of inappropriate secretion of antidiuretic hormone (SIADH), the most frequent adverse events (AEs) were thirst (16%), dry mouth (13%) and increased urination (11%) (Berl et al., 2010; Estilo et al., 2021). A primary safety concern for hyponatremia treatment is rapid adjustment of serum Na^+^ concentrations, which can elicit central pontine myelinolysis, causing severe neurological symptoms and potential death (Malhotra et al., 2014). To avoid this condition, some physicians have been reported to split or crush the tolvaptan tablets to create a 7.5-mg starting dose, believing that a lower initial dose may reduce these risks (Yuan et al., 2019).

Tolvaptan, classified as a Class 2 compound in the Biopharmaceutical Drug Disposition Classification System due to its poor solubility and substantial metabolism, undergoes primary enzymatic breakdown by the human hepatic cytochrome P-450 3A4 (CYP3A4), as indicated by *in vitro* studies (Benet, 2013). This conclusion was reinforced by a drug-drug interaction investigation in healthy individuals combining tolvaptan with the CYP3A4 inhibitor ketoconazole and inducer rifampin (Shoaf et al., 2012a). Notably, its two metabolites have been reported to be biologically inactive (Woodhead et al., 2017).

Detailed investigations of the PK and PD parameters of tolvaptan have been carried out in Caucasian, Japanese and Korean populations (Kim et al., 2011; Yi et al., 2012) and Chinese patients with child-Pugh B cirrhosis (Liu et al., 2024), but no such study has been conducted in Chinese healthy subjects. The current parallel phase 1 clinical trials were designed to elucidate the PK and PD profiles of tolvaptan in healthy Chinese subjects. Additionally, a previously published dose-finding study indicated that a 7.5 mg dose of tolvaptan may be just as effective as 15 mg and 30 mg in patients with liver cirrhosis and hepatic edema (Wang et al., 2018). As a result, the trial also included an evaluation of the lower 7.5 mg dose of tolvaptan in healthy Chinese subjects following single and multiple dose administration.

## 2 Methods

### 2.1 Study designs

Study 1 (Protocol 156–06-801–01) involved an open-label PK study of tolvaptan tablets in healthy Chinese participants that included both single and multiple dose administration. For single doses, subjects received tolvaptan orally at doses of 15, 30, 60 and 120 mg, separately, under fasting conditions. Plasma samples were collected at pre-dose as well as at 0.5, 1, 1.5, 2, 3, 4, 6, 8, 12, 24, 36, 48, 72, 96, 120 and 144 h post-dose. For multiple doses, subjects were orally administered 30 mg or 60 mg tolvaptan on days 1, 3, 4, 5, 6, 7, 8 and 9 under fasting conditions. Plasma samples were collected at pre-dose and at 0.5, 1, 1.5, 2, 3, 4, 6, 8, 12, 24, 36 and 48 h after drug administration on day 1 and 9. Plasma samples were also collected at pre-dose on days 3–8. Furthermore, the study 2 (Protocol 156–11-807–01) was a single-dose PK study of tolvaptan tablets in healthy Chinese subjects who were orally administered a single 7.5 mg dose of tolvaptan under fasting condition, with plasma samples collected at pre-dose, and then at 0.5, 1, 1.5, 2, 3, 4, 6, 8, 12, 24, 36, 48, 72, 96, 120 and 144 h after drug administration. Finally, study 3 (Protocol 156–11-808–01) was a multiple dose (7.5 mg) PK study of tolvaptan tablets in which healthy Chinese subjects were orally administered 7.5 mg of tolvaptan on days 1, 3, 4, 5, 6, 7, 8 and 9 under fasting conditions, with plasma samples collected at pre-dose, 0.5, 1, 1.5, 2, 3, 4, 6, 8, 12, 24, 36 and 48 h after drug administration on days 1, 4, 5. 6, 7, 8 and 9.

For Studies 1, 2 and 3, healthy Chinese male subjects aged 20–45 years were enrolled if their weight was >50 kg, body mass index (BMI) 19–24 kg/m^2^ and had provided signed informed consent. To be included, subjects had to be healthy, according to their medical histories, physical examinations, vital signs and routine clinical laboratory tests. Serum electrolytes, urine volume and fluid intake volumes were measured at the prescribed time points. AEs were continuously monitored and carefully documented during the studies.

Study 1 took place at Fuwai Heart Hospital, while studies 2 and 3 were carried out at Peking Union Medical College Hospital. All three studies were sponsored by Zhejiang Otsuka Pharmaceutical Co., Ltd. and carried out in compliance with Chinese Good Clinical Practice and the Declaration of Helsinki. The studies involving human participants were reviewed and approved by the institutional review board of the Peking Union Medical College Hospital (Approval number: PUMCH1178/PUMCH1179) and Fuwai Heart Hospital (Approval number: 79). The patients/participants provided their written informed consent to participate. Studies 1, 2 and 3 were registered with clinicaltrials.gov (identifier NCT07166796, NCT07166783 and NCT07166887).

### 2.2 Bioassay

Plasma concentrations of tolvaptan and its two primary metabolites, DM-4103 and DM-4107, were measured using a validated high-performance liquid chromatography-tandem mass spectrometry (HPLC-MS/MS) method using an Agilent 1100 HPLC (Agilent Technologies Inc., United States) and API4000 LC-MS instrument (Applied Biosystems, United States). Concentrations of the principal metabolites were specifically measured in healthy subjects after single-dose administration (in the single-dose part of Study 1 and Study 2). Metabolite concentrations were not analyzed in healthy subjects after multiple-dose administration (in the multiple-dose parts of Study 1 and Study 3).

The plasma sample (0.1 mL) was mixed with 0.15 mL precipitant (acetonitrile solution, containing internal standard) by vortex for 1 min, centrifuged at 10,800 rpm for 5 min, and 0.1 mL of the supernatant was separated, mixed with 1 mL phosphate buffer, and added to the activated solid phase extraction (SPE) column. Subsequently, the SPE column was washed with 1 mL pure water, primary eluent (methanol: water = 85:15), and final eluent (acetonitrile: water = 60:40), respectively. The fraction of the final eluent was collected and transferred to the autosampler vial, and a 10 μL sample solution was injected into HPLC-MS/MS for analysis. The analytes were chromatographically separated by Waters Nova-Pak C18 150 mm × 3.9 mm (5 μm) at 35 °C with the mobile phase containing acetonitrile/water/formic acid (65:35:0.25, v/v/v) at a flow rate of 0.8 mL/min under isocratic conditions. MS was operated in a positive ion mode using electrospray ionization (Turbo IonSpray). Multiple reaction monitoring of analytes was conducted in the positive mode at m/z 449.3 to 252.1, 479.2 to 252.1, 481.2 to 252.1, and 463.2 to 266.1 for tolvaptan, DM-4103, DM-4107 and the internal standard, respectively. The retention times of tolvaptan, DM-4103, DM-4107 and internal standard were 2.27, 2.69, 1.75 and 2.44 min, respectively. The standard curve was fitted by analyte-to-internal standard peak area ratios, using weighted least-squares regression analysis, and R was 0.9994, 0.9994 and 0.9998 for tolvaptan, DM-4103 and DM-4107, respectively. The linear range of calibration curve for tolvaptan, DM-4103 and DM-4107 were 1–500 ng/mL, with the lower limits of quantification (LLOQ) being 1 ng/mL. The stability evaluation included the stability of plasma samples stored at 4 °C for 76 h and repeated freeze-thaw 3 times, and the stability of tolvaptan, DM-4103 and DM-4107 was acceptable. The deviation accuracy of tolvaptan, DM-4103 and DM-4107 were 4.1∼8.1%, 3.7∼10.3% and 4.1∼10.7%, respectively.

### 2.3 Pharmacokinetics and dose proportionality

The non-compartmental PK analysis was carried out using Phoenix WinNonlin ver. 6.1. In the single-dose phases of Study 1 and Study 2; PK parameters, including AUC, C_max_, time to reach C_max_ (t_max_), CL/F, and t_1/2_, were calculated for tolvaptan.

All five dose levels (7.5, 15, 30, 60 and 120 mg) were included in the evaluation of dose proportionality after a single-dose. Dose proportionality was evaluated using a power model; (Gough et al., 1995); if the 95% confidence interval (CI) of r^β−1^ included 1, dose proportionality was confirmed within the corresponding dose range. Otherwise, dose proportionality was not established. Similarly, dose proportionality was also evaluated after the first and last dose in healthy subjects in Study 1 and Study 3.

### 2.4 PD assessment

For PD analyses, absolute values were measured for the cumulative urine output at 24 h, the volume of fluid intake, and fluid balance (FB) where FB = urine volume - fluid intake volume. Urine output and fluid intake volumes were measured at intervals of 0–2 h, 2–4 h, 4–6 h, 6–8 h, 8–12 h, 12–24 h and 24–48 h after single-dose administration, as well as after the first and last dose in the multiple-dose study. Serum electrolytes (K^+^, Na^+^, Cl^−^, Mg^2+^) were measured before tolvaptan was given and at 2, 4, 6, 8, 12, 24 and 48 h after a single dose. They were also measured at 2, 4, 6, 8, 12 and 24 h on Day1 and Day 9, and 6 h after administration on days 4 and 6 in the multiple dose study.

### 2.5 Statistical analysis

PD endpoints together with changes from baseline as well as PK parameters are presented using descriptive statistics according to time points or collection intervals and the tolvaptan dose using SPSS ver. 26.0. PK parameters were calculated using non-compartmental modeling methods by WinNonlin (ver. 6.1) software. Slopes and 95% CIs of the plots of individual subject log dose vs. log C_max_ or log AUC  ∞ were evaluated using a Power Model method.

## 3 Results

### 3.1 Participant characteristics

**FIGURE 1:**
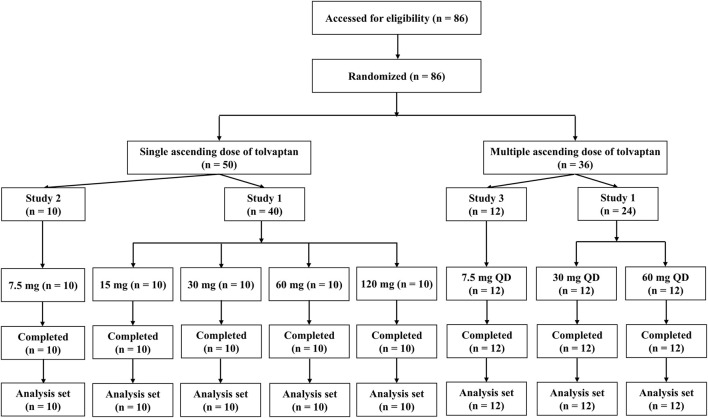
Flowchart of subject disposition. QD, once a day.

A total of 86 male healthy subjects were enrolled in the three clinical trials and all subjects completed the trials (Figure 1). Descriptive statistics for demographics, including age, and body weight are shown in Supplementary Table S1. Demographic characteristics were consistent across dose levels and trial sites.

### 3.2 Pharmacokinetics and dose proportionality

**FIGURE 2:**
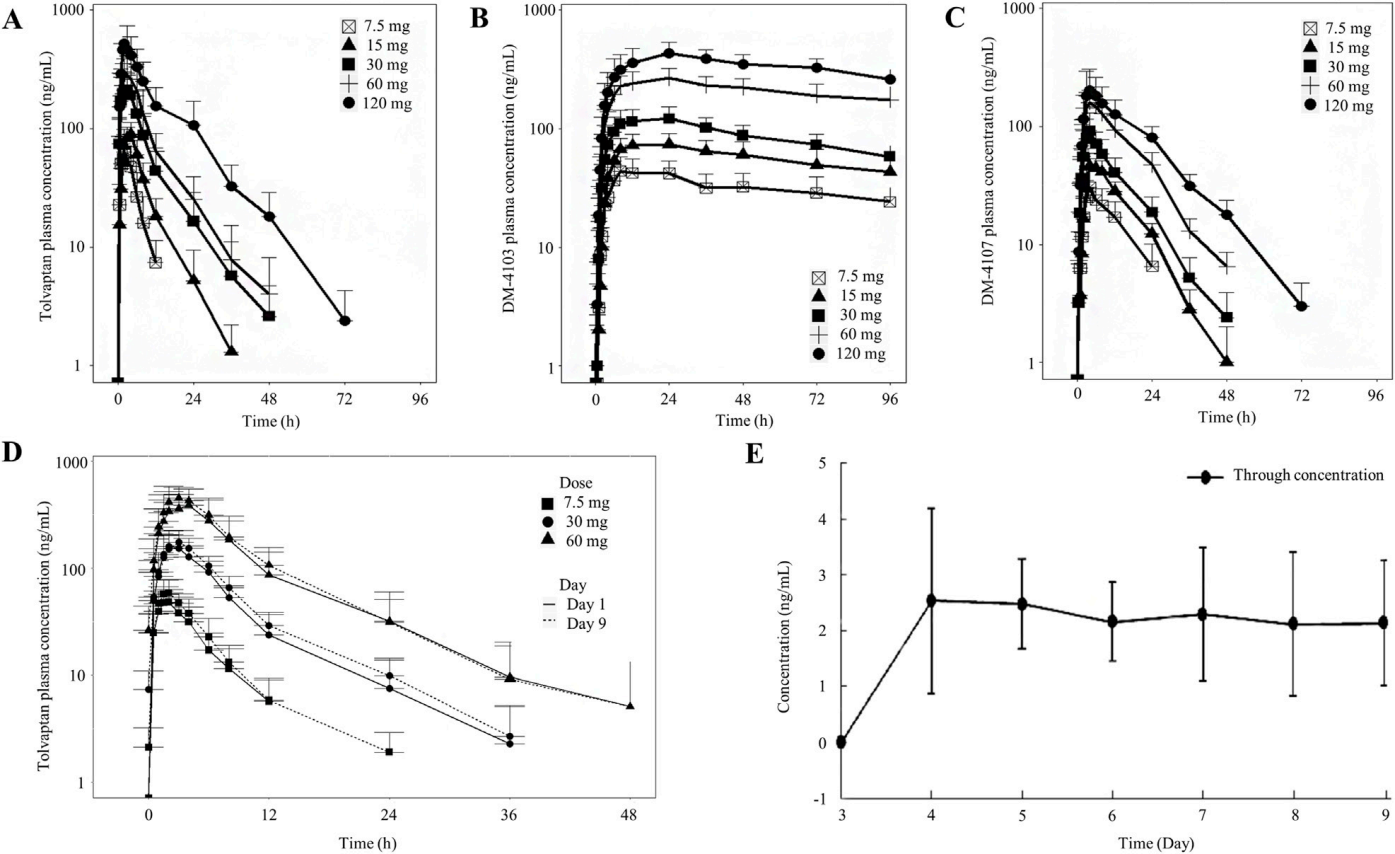
Mean (SD) tolvaptan concentration time profiles. **(A–C)** After single dose oral administration of 7.5, 15, 30, 60 and 120 mg; **(D,E)** On Day 1 and Day 9 after oral administration of 7.5, 30 and 60 mg.

Descriptive statistics for various PK parameters after a single-dose or multiple-doses of tolvaptan are presented in Tables 1, 2, along with the corresponding dose proportionality assessment results. In addition, tolvaptan concentration-time profiles at 24, 48, 72 and 96 h after single dose oral administration of 7.5, 15, 30, 60 and 120 mg are presented in Figures 2A–C. The time-dependent tolvaptan decreases on Day 1 and Day 9 are shown in Figures 2D,E.

**TABLE 1:** Pharmacokinetic parameters in healthy subjects after single dose administration of tolvaptan (7.5, 15, 30, 60 and 120 mg).

PK parameters	Study 2	Study 1 (single dose)				Dose proportionality
	7.5 mg (n = 10)	15 mg (n = 10)	30 mg (n = 10)	60 mg (n = 10)	120 mg (n = 10)	Estimate (95% CI)
Tolvaptan						
AUC_0-t_ (ng·h/mL)	379.5 (176.1)	774.5 (195.8)	2,041.3 (834.1)	2,922.9 (1,220.8)	6,601.3 (2,300.5)	1.058 (0.763,1.467)
AUC_0-_ ∞ (ng·h/mL)	392.2 (177.5)	787.0 (196.1)	2,077.0 (852.2)	2,954.5 (1,218.2)	6,673.8 (2,263.4)	1.037 (0.752,1.430)
C_max_ (ng/mL)	69.8 (24.0)	102.0 (17.4)	245.5 (82.9)	323.9 (141.4)	587.1 (364.0)	0.495 (0.355,0.690)
t_max_ ^a^ (h)	2 (1, 4)	3 (1.5, 6)	2.5 (1, 4)	2.5 (1, 6)	2 (1.5, 6)	
t_1/2_ (h)	4.1 (2.2)	6.8 (3.4)	9.1 (2.7)	11.2 (5.6)	10.0 (3.0)	
DM-4103						
AUC_0-t_ (ng·h/mL)	4,132.6 (801.9)	7,375.6 (1,994.2)	10,773.6 (2,442.3)	27,687.0 (6,269.4)	43,391.0 (9319.8)	0.753 (0.507,1.118)
C_max_ (ng/mL)	52.0 (11.4)	75.5 (16.3)	125.9 (33.5)	270.4 (53.1)	438.0 (103.3)	0.673 (0.486,0.933)
t_max_ ^a^ (h)	18 (3, 48)	24 (8, 24)	24 (8, 24)	24 (8, 48)	24 (12, 36)	
t_1/2_ (h)		155.1 (55.8)	100.7 (16.8)	158.5 (61.6)	138.5 (48.1)	
DM-4107						
AUC_0-t_ (ng·h/mL)	374.6 (171.2)	737.3 (151.1)	1,253.4 (382.3)	2,748.7 (554.9)	4,210.6 (1,212.7)	0.773 (0.592,1.009)
C_max_ (ng/mL)	32.0 (10.8)	48.7 (11.0)	95.0 (35.6)	167.0 (43.6)	210.1 (103.5)	0.442 (0.327,0.597)
t_max_ ^a^ (h)	4 (3, 8)	5 (4, 8)	4 (3, 6)	4 (3, 8)	4 (3, 6)	
t_1/2_ (h)	6.5 (2.2)	7.5 (1.1)	10.0 (3.7)	10.4 (4.0)	10.1 (1.8)	

Data are presented as the mean (SD) unless otherwise specified.

^a^ For T_max_, Median (Min-Max).

**TABLE 2:** Pharmacokinetic parameters in healthy subjects after multiple doses of tolvaptan (7.5, 30 and 60 mg).

PK parameters	Study 3		Study 1 (multiple doses)				Dose proportionality	
	7.5 mg QD (n = 12)		30 mg QD (n = 12)		60 mg QD (n = 12)			
	First dose	Last dose	First dose	Last dose	First dose	Last dose	First dose	Last dose
t_1/2_ (h)	4.7 (2.1)	4.6 (1.3)	7.1 (2.2)	6.6 (1.8)	7.5 (2.9)	7.1 (4.0)		
t_max_ (h)^a^	2 (0.5, 3.0)	2 (1.0, 4.0)	2.5 (1.5, 6.0)	3 (1.5, 6.0)	3.0 (1.0, 6.0)	2.5 (1.0, 4.0)		
C_max_ (ng/mL)	53.9 (18.6)	64.5 (15.4)	182.6 (45.6)	189.8 (46.5)	459.5 (181.4)	522.1 (121.3)	1.161 (0.594, 2.269)	1.372 (0.402, 4.688)
AUC_0–24h_ (ng·h/mL)	290.4 (109.6)	341.4 (99.9)	1181.7 (172.0)	1354.7 (218.9)	3469.7 (1405.7)	3999.9 (1120.8)	1.735 (0.832, 3.618)	1.995 (0.802, 4.963)
AUC_0-_ ∞ (ng·h/mL)	303.7 (122.0)	352.1 (107.0)	1271.4 (196.1)	1461.3 (252.5)	3878.8 (1583.6)	4413.7 (1417.0)		
CL/F (L/h)	28.6 (10.9)	23.9 (7.6)	24.1 (3.6)	22.6 (3.3)	17.6 (6.4)	16.0 (4.1)		
C_avg_ (ng/mL)	-	14.2 (4.2)	-	56.5 (9.1)	-	166.7 (46.7)		
C_min_ (ng/mL)	-	1.9 (1.0)	-	7.2 (3.5)	-	25.0 (16.8)		
R_pred_ ^b^	1.1 (0.1)	-	1.1 (0.1)	-	1.1 (0.2)	-		
R_ac_ ^c^	-	1.2 (0.3)	-	1.2 (0.2)	-	1.2 (0.4)		

Data are presented as the mean (SD) unless otherwise specified.

^a^ T_max_, Median (Min-Max).

^b^ R_pred_ (predicted accumulation ratio) = 1/(1-exp (-Ke·τ)), where Ke is the elimination rate constant after a single dose, τis the dosing interval (24 h).

^c^ R_ac_ (observed accumulation ratio) = AUC_0-24_ (Day 7)/AUC_0-24_ (Day 1).

After a single-dose, the AUC_0-t_ and C_max_ of tolvaptan and its two principal metabolites increased with the dose. In the 7.5 mg–120 mg range, AUC_0-t_ for tolvaptan and its metabolites showed dose proportionality, while C_max_ increased less than dose proportionally. The terminal half-life extended with higher doses, and the time to reach C_max_ was consistent across all doses, with a median of 2 h–3 h. Although the 90% CIs for the AUC_0-t_ of DM-4103 and DM-4107 included 1, both AUC_0-t_ and C_max_ showed less than dose proportional increases from 7.5 mg to 60 mg. Moreover, following multiple dosing, study participants reached steady-state by Day 7, demonstrating that the C_max_ and AUC_0-t_ of tolvaptan increased with dose. AUC_0-t_ increased more than dose proportionally in the range of 7.5 mg–60 mg, whereas C_max_ showed dose proportionality.

### 3.3 PD results

Subjects given a single dose of tolvaptan had a significant increase in their 24 h urine output and fluid intake. The 24 h urine volumes after single doses of 7.5, 15, 30, 60 and 120 mg tolvaptan were 4,589.5 ± 1,400.7 mL, 4,497.0 ± 1,640.1 mL, 7,775.0 ± 1,711.5 mL, 9,439.0 ± 2,463.4 mL and 12,447.0 ± 2,578.2 mL, respectively (Table 3). Both the urine volume and the fluid intake were increased in a manner that was dose-dependent. Although the urine output minus fluid intake was negative, any differences in the various tolvaptan dose groups were not statistically significant. After multiple doses of tolvaptan, the 24 h urine output was also increased in a dose-dependent manner, though urine output after the last dose was less than after the first.

**TABLE 3:** 24 h-urine volume and water intake in healthy male subjects after single and multiple oral doses of tolvaptan tablets.

PD indicators	Single dose					Multiple doses					
	7.5 mg (n = 10)	15 mg (n = 10)	30 mg (n = 10)	60 mg (n = 10)	120 mg (n = 10)	7.5 mg QD (n = 12)		30 mg QD (n = 12)		60 mg QD (n = 12)	
						First dose	Last dose	First dose	Last dose	First dose	Last dose
Urine output, mL	4,589.5 ± 1,400.7	4,497.0 ± 1,640.1	7,775.0 ± 1,711.5	9,439.0 ± 2,463.4	12,447.0 ± 2,578.2	3,993.4 ± 734.2	3,432.1 ± 916.9	7,738.3 ± 1,209.0	6,680.0 ± 1,390.5	10,127.5 ± 2,439.8	7,235.0 ± 1,356.1
Fluid intake, mL	3,450.0 ± 1,474.6	3,713.0 ± 1,522.5	6,240.5 ± 1,246.1	7,899.0 ± 1,718.3	9,477.5 ± 1,584.0	2,654.2 ± 735.3	2,600.0 ± 977.2	9,759.2 ± 2,263.9	9,095.4 ± 1,732.0	12,327.9 ± 2,093.6	9,763.7 ± 1,458.0

**FIGURE 3:**
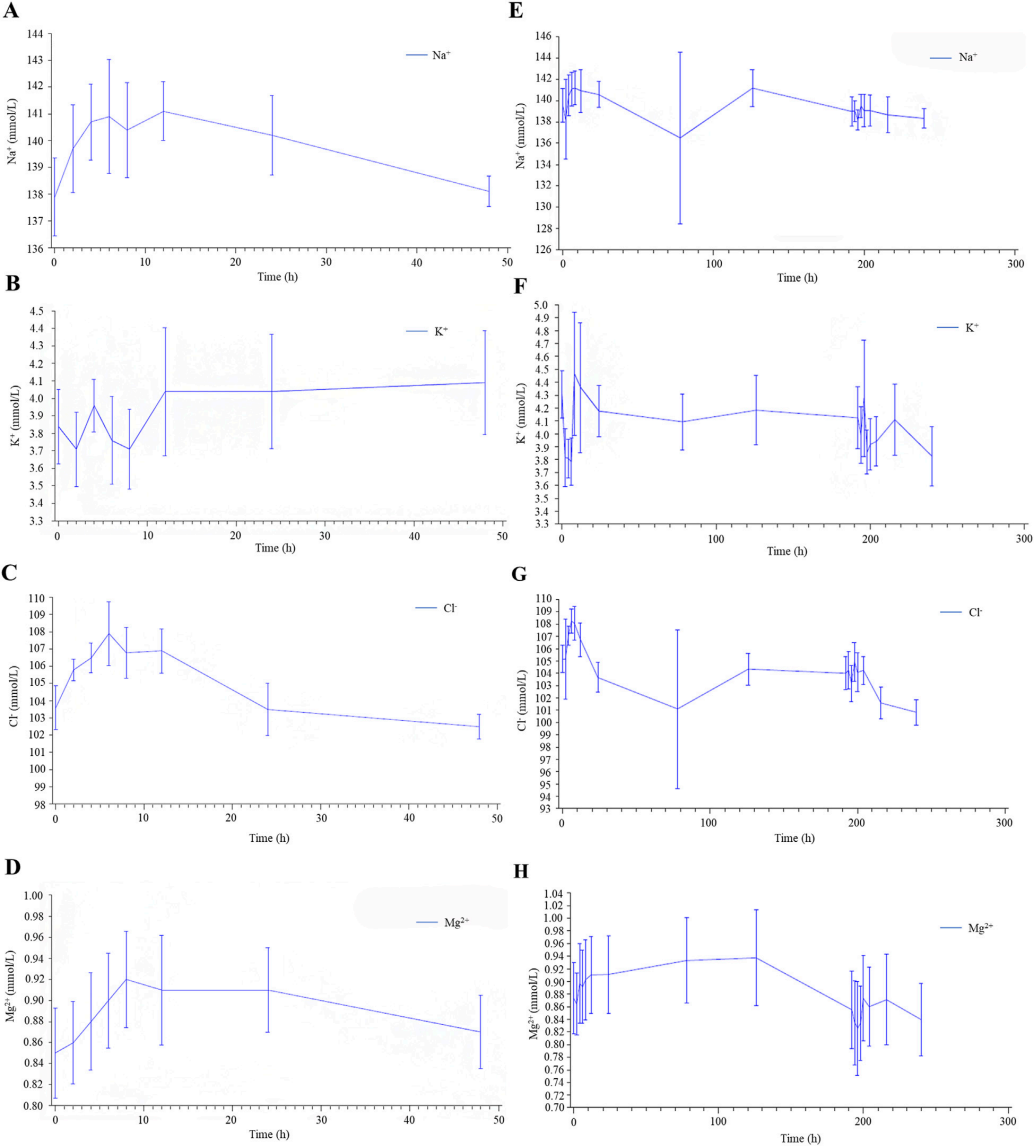
Serum electrolyte results in healthy male subjects after single **(A–D)** and multiple **(E–H)** oral doses of tolvaptan tablets.

With the exception of one subject who experienced a mild reduction in serum Na^+^ during the multiple administrations of 7.5 mg tolvaptan, no clinically significant abnormalities in blood electrolytes (K^+^, Na^+^, Cl^−^, Ca^2+^) were observed in the other subjects (Figure 3).

### 3.4 Adverse events

A total of 15 treatment-emergent adverse events (TEAEs) were reported in 12 of 50 subjects following single-dose administration of tolvaptan at doses ranging from 7.5 mg to 120 mg, while 22 TEAEs were reported in 16 of 36 subjects following multiple-dose administration of tolvaptan at doses ranging from 7.5 mg to 60 mg once daily for 7 days. The most frequently reported single-dose TEAEs were thirst (n = 11 (22%) of 50 subjects) and dry mouth (n = 3 (6.0%) of 50 subjects. Following multiple doses, the most commonly reported TEAEs were dry mouth (n = 8 (22.2%) of 36 subjects and thirst (n = 3 (8.3%) of 36 subjects. These adverse events were attributed to the pharmacological action of tolvaptan as an aquaretic. All reported TEAEs were of mild severity and no serious adverse events were observed. The incidence of TEAEs is detailed in Table 4.

**TABLE 4:** Summary of treatment-emergent adverse events after single and multiple oral doses of tolvaptan.

System organ class	Preferred term	Single dose						Multiple dose			
		7.5 mg (n = 10)	15 mg (n = 10)	30 mg (n = 10)	60 mg (n = 10)	120 mg (n = 10)	Total (n = 50)	7.5 mg QD (n = 12)	30 mg QD (n = 12)	60 mg QD (n = 12)	Total (n = 36)
Gastrointestinal disorders, n (%)	Dry mouth	-	-	-	-	3 (30.0)	3 (6.0)	1 (8.3)	2 (16.7)	5 (41.7)	8 (22.2)
	Thirst	-	5 (50.0)	-	3 (30.0)	3 (30.0)	11 (22.0)	-	-	3 (25.0)	3 (8.3)
	Pharyngeal discomfort	-	-	1 (10.0)	-	-	1 (2.0)	-	-	-	0
Investigations n (%)	Blood bilirubin increased	-	-	-	-	-	0	2 (16.7)	-	-	2 (5.6)
	Blood CK level increased	-	-	-	-	-	0	1 (8.3)	-	1 (8.3)	2 (5.6)
	Blood Na^+^ decreased	-	-	-	-	-	0	1 (8.3)	-	-	1 (2.8)
	Blood uric acid increased	-	-	-	-	-	0	3 (25.0)	-	-	3 (8.3)

CK, creatine phosphokinase.

## 4 Discussion

The present study included three separates phase 1 trials that examined the PK and PD of orally administered tolvaptan in healthy Chinese male individuals. These trials were conducted in an outpatient setting at a single caregiving facility without mandated fluid restriction, which demonstrated that after a single dose, dose proportionality was observed for the AUC from 7.5 mg to 120 mg, but not for C_max_. The mean (SD) C_max_ values for the 7.5, 15, 30, 60 and 120 mg doses were 69.8 (24.0), 102.0 (17.4), 245.5 (82.9), 323.9 (141.4), and 587.1 (364.0) ng/mL, respectively. The mean (SD) AUC_0-t_ values were 379.5 (176.1), 774.5 (195.8), 2,041.3 (834.1), 2,922.9 (1,220.8) and 6,601.3 (2,300.5) hr*ng/mL, respectively. Furthermore, dose proportionality for AUC was observed with steady-state achieved within 7 days after multiple doses of 7.5, 30 and 60 mg once daily for 7 days. Furthermore, the study showed that urine volume increased with dose after both single and multiple oral administrations of tolvaptan within a dose range 7.5–120 mg.

Earlier studies established that the minimum effective concentrations of tolvaptan were reached at a serum concentration of approximately 25 ng/mL, with a maximal increase in urine output measured when tolvaptan concentrations exceeded 100 ng/mL. Interestingly, for single doses ranging from 60 to 480 mg, 0–12 h urine volumes remained comparable, suggesting saturation of effect for the 60 mg dose for up to 12 h post-administration. In a previous report, mean peak serum concentrations following a 15 mg dose were found to be 135.0 ± 53.0 ng/mL and 103.5 ± 39.5 ng/mL (Kim et al., 2011; Yi et al., 2012; Bhatt et al., 2014). These findings indicated an expected dose-dependent relationship in terms of free water clearance and urine volumes for doses ranging from 3.75 mg to 15 mg (Bondanelli et al., 2023). In the trial involving healthy adults, a dose-dependent response was evident, likely due to all individuals receiving all three doses, thereby minimizing subject variability.

Furthermore, earlier studies revealed that tolvaptan did not accumulate in either healthy subjects or patients with hepatocirrhosis following multiple dose administration (Cárdenas et al., 2012; Liu et al., 2024), which was consistent with the single-dose PK findings predicting an accumulation ratio as shown in Table 2. Dose proportionality was assessed after both single and multiple tolvaptan administration in healthy subjects. Similar to observations in healthy Korean subjects, tolvaptan AUC_0-t_ increased proportionally with the administered dose, whereas C_max_ did not increase in proportion. The CL/F of tolvaptan ranged from 22.7 ± 9.6 L/h to 19.8 ± 6.1 L/h for single doses between 7.5 mg and 120 mg, signifying dose-independent CL/F across this range. Additionally, the terminal half-life (t_1/2_) increased from approximately 4 h at 7.5 mg to 10 h at 120 mg. Following administration of multiple doses from 7.5 mg once a day to 60 mg once a day, an increase in CL/F was also observed. Moreover, in a comparison between healthy Chinese participants and their counterparts from Korea, Japan and Caucasians, after a single dose of tolvaptan (Shoaf et al., 2007; Kim et al., 2011; Shoaf et al., 2012b; Yi et al., 2012; Shoaf et al., 2017), Chinese subjects exhibited a 50% higher AUC_0-t_ and C_max_ at the corresponding dose level compared to Korean subjects. Conversely, while Chinese subjects had a similar C_max_, they showed a 30% higher AUC_0-t_ compared to Caucasian and Japanese subjects after a single 30 mg dose, as shown in Table 5.

**TABLE 5:** Pharmacokinetic parameters comparison across different populations.

Tolvaptan dose	Population	C_max_ (ng/mL)	AUC_0-24_ (ng·h/mL)	N	Study ID
**15 mg**	Chinese 1	102.0 ± 17.4	774.5 ± 195.8	10	156–06-801–01
	Korean	103.5 ± 39.5	467.1 ± 178.8	6	156-KOA-0801 (Yi et al., 2012)
	Japanese	135.0 ± 53.0	698.0 ± 375.0	6	156–00-001 (Kim et al., 2011)
**30 mg**	Chinese 1	245.5 ± 82.9	2,041.3 ± 834.1	10	156–06-801–01
	Chinese 2	182.6 ± 45.6	1,181.7 ± 172.0	12	156–06-801–01
	Japanese	213.0 ± 76.0	1,385 ± 559	12	156–00-001 (Kim et al., 2011)
	Korean	190.5 ± 45.6	1,281.2 ± 432.9	24	156-KOA-0801 (Yi et al., 2012)
**60 mg**	Chinese 1	323.9 ± 141.4	2,922.9 ± 1,220.8	10	156–06-801–01
	Chinese 2	459.5 ± 181.4	3,469.7 ± 1,405.7	12	156–06-801–01
	Japanese	315.0 ± 105.0	2,424.0 ± 606.0	6	156–00-001 (Kim et al., 2011)
	Korean	247.7 ± 65.8	1,911.7 ± 642.8	6	156-KOA-0801 (Yi et al., 2012)
	American	450.0 ± 168.0	3,660.0 ± 1,390.0	6	156–98-210 (Shoaf et al., 2007)
**120 mg**	Chinese 1	587.1 ± 364.0	6,601.0 ± 2,300.5	10	156–06-801–01
	Japanese	661.0 ± 276.0	6,071.0 ± 2,097.0	6	156–00-001 (Kim et al., 2011)
	American	564.0 ± 124.0	5,800.0 ± 1,640.0	6	156–98-210 (Shoaf et al., 2007)

Bold means data are presented as the mean ± SD.

After administering single and multiple doses of tolvaptan to healthy Chinese subjects, a notable, dose-proportional augmentation in urine volume was detected. This response was consistent with tolvaptan’s recognized pharmacological function as an arginine vasopressin receptor antagonist. Furthermore, fluid intake escalated with higher tolvaptan doses, particularly after multiple administration, as participants experienced prolonged thirst leading to increased water consumption. Even though individuals treated with tolvaptan displayed a significant rise in fluid intake, there was not always a direct correlation with self-reported thirst, potentially indicating underreporting of the sensation.

In spite of the substantial boost in urine output, the majority of subjects did not exhibit serum electrolyte imbalances. These results align with tolvaptan’s mechanism, which triggers aquaresis by impeding water reabsorption in the renal collecting ducts without significantly affecting electrolyte excretion (Zmily et al., 2011). The observed elevation in urine volume without a concurrent electrolyte disruption accentuates tolvaptan’s specific diuretic impact. This mechanism ensures efficient fluid clearance while upholding electrolyte stability, positioning tolvaptan as a potent therapeutic choice for conditions necessitating heightened urine output without the risk of electrolyte disturbances (Tzoulis et al., 2023). Tolvaptan proficiently enhances urine volume and fluid intake in a dose-dependent manner without inducing substantial serum electrolyte imbalances, reaffirming its pharmacological characteristics and clinical value (Fukuoka et al., 2020).

Post-marketing evaluations in Europe have revealed a practice among physicians of splitting or crushing tolvaptan tablets to create a 7.5 mg initial dose for the treatment of hyponatremia to potentially minimize the risk of over-rapid correction of serum Na^+^ concentrations. As no PK/PD studies have been carried out on SIADH patients with hyponatremia, a trial was undertaken to assess the initial response of serum Na^+^ concentrations to various tolvaptan doses (Shoaf et al., 2017). Additionally, a prior dose-finding study suggested that a 7.5 mg dose could be as efficacious as higher doses in patients with liver cirrhosis and hepatic edema (Wang et al., 2018), prompting an evaluation of the lower 7.5 mg dose in healthy Chinese subjects following both single and multiple administration of tolvaptan. Furthermore, early investigations into the PK/PD characteristics of 7.5 mg tolvaptan in SIADH patients found that starting titration with 7.5 mg did not eliminate the risk of rapid serum Na^+^ corrections, with fluid balance monitoring potentially identifying individuals at risk of overcorrection (Tzoulis et al., 2023). The trial focusing on the PK/PD of 7.5 mg tolvaptan demonstrated no clinically significant alterations in serum electrolytes (K^+^, Na^+^, Cl^−^, Mg^2+^) following oral administration of a 7.5 mg dose. The most frequently reported AEs after single and multiple doses of 7.5 mg tolvaptan were thirst, dry mouth and pharyngeal discomfort, which are anticipated side effects of tolvaptan. Collectively, these findings support the effectiveness of 7.5 mg tolvaptan with a reduced risk of overly rapid correction of the serum Na^+^ concentration.

Inherent limitations of conducting a phase 1 study are the short duration of treatment (7 days) due to the fact that the enrolled subjects were all healthy. In addition, the open-label design of the trial may have biased the results, which is mitigated by the similar design of the three trials, and no unexpected TEAEs of tolvaptan being identified, all of which were mild in severity. Three phase 1 clinical trials have investigated the PK and PD characteristics of tolvaptan (7.5–120 mg) in healthy Chinese males, but lacked generalizations in the female population. Previous studies have reported that female healthy subjects had higher tolvaptan exposure (AUC) and lower apparent clearance than males, but given the low proportion of women in the sample size and the large inter-individual variation in most studies, it is unclear whether this reflects a gender difference in PK characteristics (Yi et al., 2012). In addition, due to the short duration of treatment and the possible differences in PK/PD profiles between healthy subjects and patients with liver/renal insufficiency, PK/PD results should be interpreted with caution.

In conclusion, tolvaptan was found to be safe and well-tolerated by healthy Chinese male subjects after single dose and multiple dose administration. Both single and multiple doses of tolvaptan led to a dose-dependent increase in the volume of urine. Dose proportionality was observed in AUC but not in C_max_ after single dose administration, ranging from 7.5 mg to 120 mg. Exposure levels following single-dose administration in Chinese male subjects were comparable to those in Japanese and Caucasian individuals but higher than in their Korean counterparts.

## Data Availability

The original contributions presented in the study are included in the article/Supplementary Material, further inquiries can be directed to the corresponding author.
