# Supplementary material for: Clinical trial on the pharmacokinetics, pharmacodynamics and safety of tolvaptan in healthy Chinese males: an open-label, single and multiple dosage, parallel group study
Source: Front Pharmacol. 2025 Nov 21;16:1713702. doi: 10.3389/fphar.2025.1713702 (PMC12679040; doi:10.3389/fphar.2025.1713702)
Supplement: Supplementary file 1 [file Table1.pdf]

Supplementary Table 1. Demographic characteristics of subjects enrolled in the studies

Items	Study 1						Study 2	Study 3
	15 mg (n = 10)	30 mg (n = 10)	60 mg (n = 10)	120 mg (n = 10)	30 mg QD (n = 12)	60 mg QD (n = 12)	7.5 mg (n = 10)	7.5 mg QD (n = 12)
Age (years)	22.9 ± 1.4	24.5 ± 2.4	22.9 ± 1.0	24.4 ± 3.7	23.3 ± 2.3	23.5 ± 2.9	26.9 ± 3.0	25.1 ± 2.4
Weight (kg)	63.4 ± 5.3	63.4 ± 4.7	64.6 ± 6.8	63.5 ± 6.7	62.0 ± 5.9	61.2 ± 7.4	61.2 ± 5.0	62.9 ± 6.4
BMI (kg/m^2)	22.4 ± 1.1	21.4 ± 1.3	22.4 ± 1.3	22.3 ± 1.4	21.3 ± 1.5	21.3 ± 1.7	21.7 ± 1.7	21.8 ± 1.6

Data are presented as the mean ± SD unless otherwise specified.

Abbreviations: BMI, body mass index; QD, once a day; SD, standard deviation
